# 1000 human genomes carry widespread signatures of GC biased gene conversion

**DOI:** 10.1186/s12864-018-4593-1

**Published:** 2018-04-16

**Authors:** Rajib Dutta, Arnab Saha-Mandal, Xi Cheng, Shuhao Qiu, Jasmine Serpen, Larisa Fedorova, Alexei Fedorov

**Affiliations:** 1 Program in Biomedical Sciences, University of Toledo, Health Science Campus, Toledo, OH 43614 USA; 2 Department of Medicine, University of Toledo, Health Science Campus, Toledo, OH 43614 USA; 3 Program in Bioinformatics and Proteomics/Genomics, University of Toledo, Health Science Campus, Toledo, OH 43614 USA; 4 SURF Program, University of Toledo, Health Science Campus, Toledo, OH 43614 USA; 5 GEMA-biomics, Ottawa Hills, Lucas County, OH 43606 USA; 6 Present Address: Center for Cardiovascular and Pulmonary Research, Nationwide Children’s Hospital, 700 Children’s Dr, Columbus, OH USA; 7 Present Address: Biochemistry and Molecular Biology Graduate Program, Cumming School of Medicine, University of Calgary, Calgary, AB T2N4N1 Canada; 8 College of Arts and Sciences, Washington University in St. Louis, 1 Brookings Dr, St. Louis, MO 63130 USA

**Keywords:** SNP, Polymorphism, Bioinformatics, Mutation, Evolution

## Abstract

**Background:**

GC-Biased Gene Conversion (gBGC) is one of the important theories put forward to explain profound long-range non-randomness in nucleotide compositions along mammalian chromosomes. Nucleotide changes due to gBGC are hard to distinguish from regular mutations. Here, we present an algorithm for analysis of millions of known SNPs that detects a subset of so-called “SNP flip-over” events representing recent gBGC nucleotide changes, which occurred in previous generations via non-crossover meiotic recombination.

**Results:**

This algorithm has been applied in a large-scale analysis of 1092 sequenced human genomes. Altogether, 56,328 regions on all autosomes have been examined, which revealed 223,955 putative gBGC cases leading to SNP flip-overs. We detected a strong bias (11.7% ± 0.2% excess) in AT- > GC over GC- > AT base pair changes within the entire set of putative gBGC cases.

**Conclusions:**

On average, a human gamete acquires 7 SNP flip-over events, in which one allele is replaced by its complementary allele during the process of meiotic non-crossover recombination. In each meiosis event, on average, gBGC results in replacement of 7 AT base pairs by GC base pairs, while only 6 GC pairs are replaced by AT pairs. Therefore, every human gamete is enriched by one GC pair. Happening over millions of years of evolution, this bias may be a noticeable force in changing the nucleotide composition landscape along chromosomes.

**Electronic supplementary material:**

The online version of this article (10.1186/s12864-018-4593-1) contains supplementary material, which is available to authorized users.

## Background

One of the longstanding questions in mammalian genome evolution has been the origin of GC-isochors, which are long (> 100 kb) chromosome segments characterized by a high degree of uniformity in GC-composition levels [1, 2]. Several theories including selectionism [3], neutralism [4], thermodynamic stability [5], and GC-Biased Gene Conversion (gBGC) [6] have been proposed to explain the origin of isochors, but have not been conclusive. The gBGC hypothesis was initially formulated by Holmquist [7] and Eyre-Walker [8, 9], and has been elaborated upon since then [10, 11]. gBGC is proposed to be a consequence of special cases of meiotic recombinations that involve the formation of heteroduplexes [11, 12]. A heteroduplex is created when a short single-stranded DNA segment of one of the parental chromosomes forms a double stranded structure with its complementary homologous strand from the equivalent chromosome of another parent. The presence of SNPs within a heteroduplex results in mismatched base pairs that are resolved by the molecular machinery of the DNA mismatch repair (MMR) pathway [13]. gBGC hypothesizes a bias in the repair, in which mismatched nucleotide pairs are resolved in favor of G-C pairs [10]. This would imply that mismatches involving non-Watson-Crick base pairs such as A-G, T-C, A-C or T-G would be preferentially repaired to yield C-G, G-C, G-C and C-G Watson-Crick base pairs respectively. In 2013, Lesecque and co-authors experimentally confirmed the existence of such bias in yeast, yet it appeared to be very small (50.6% vs 49.4% in AT - > GC base pair changes vs. GC - > AT ones) [14].

One important consequence of gBGC is the increased overall GC-content at recombination hotspots [15], where heteroduplexes are more frequent than elsewhere. There are both supporting and opposing evidences for gBGC. The supporting propositions posit that gBGC explains the evolution of non-randomness in GC-compositions within mammalian genomes [11], the rapid fixation of AT- > GC mutations [11, 16] and increased GC content of recombining DNA in mammals and yeast [17]. Evidence of gBGC has also been reported in yeasts [18], arabidopsis [19], and honeybee genomes [20]. On the other hand, conflicts with the gBGC hypothesis have also been published. For example, analysis of GC/AT and AT/GC substitutions in the human Fetuin-A gene ruled out gBGC as one of the causal factors [21]. Population genomic analysis of *Drosophila melanogaster* revealed no evidence for gBGC [22] and, in another instance, non-allelic gene conversion processes in Drosophila and primate genomes also negated the contribution of gBGC towards organism diversity [23]. A negative correlation between substitution and recombination rates in the chicken genome was also reported, inconsistent with the gBGC model [24]. Even though gBGC with respect to humans has been reported in recombination hotspots and rapidly-evolving regions of the human genome [25, 26], a quantitative picture of gBGC inside any region of the human genome was lacking until recently [11]. In the last 2 years, new publications reported directly observed cases of gBGC events in several large families [27, 28]. These authors demonstrated a strong bias in AT- > GC over GC- > AT base pair changes during non-crossover gene conversions in humans.

In the modern time when whole-genome sequencing is a routine and thousands of human genomes are available, is it possible to detect and quantify gBGC events in a large scale? One of the fundamental principles in genome organization is that neighboring SNPs are linked into haplotypes. A gene conversion event results in replacement of an allele inherited from one parent by the complementary allele inherited from another parent. About half of gBGC episodes occur during crossover (CO) miotic recombinations while another half during non-crossover (NCO) recombinations. In the latter case of NCO, the gBGC allele replacement occurs within the same parental haplotype producing only single nucleotide change inside this haplotype. We call this specific process a “SNP flip-over” throughout this paper. In other words, SNP flip-over is an allele replacement event for a single SNP in the middle of evolutionarily conserved haplotype. Such SNP flip-over may be directly detected by comparing the genomes of mother, father, and offspring, when haplotypes of all three are available. However, since the New Generation Sequencing technique is dependent on assembling millions of short reads, haplotypes are computationally deduced in a so-called “phasing” procedure. It is a statistical prediction that generates a vast number of phasing errors [29–31]. Phasing problems make the detection of de novo gBGC events difficult. To overcome this problem Williams with coauthors and Halldorsson with coauthors studied three-generation pedigrees with multiple family members [27, 28]. Such direct detection of gBGC is of ultimate importance for the validation of this phenomenon yet it provides limited statistics.

**Fig. 1:**
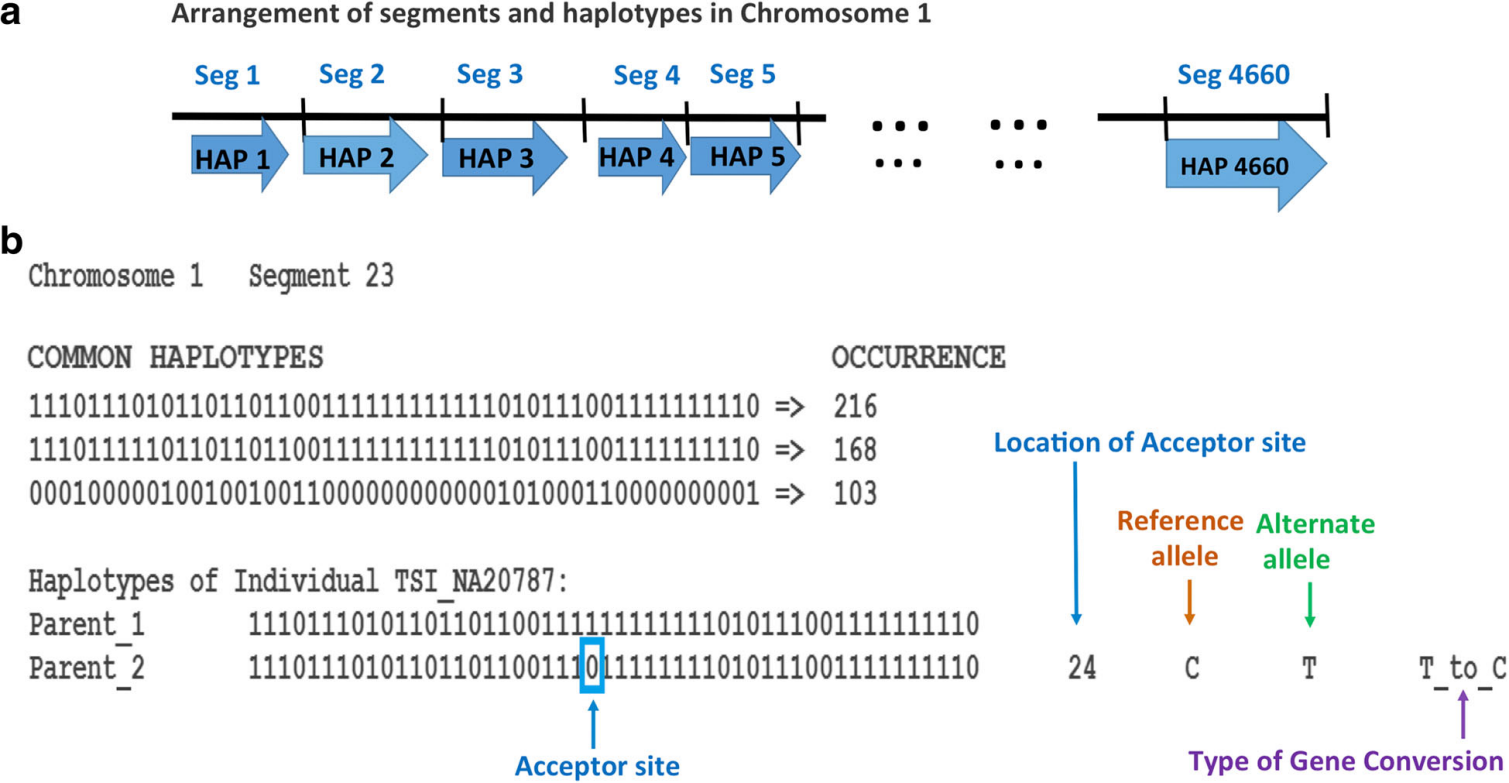
Characterization of haplotypes of frequent genetic variants and putative case of BGC event. **a** - arrangement of computationally processed chromosomal segments for analysis of haplotypes. Autosomes have been divided into 56,328 segments, each containing 50 high-frequency (MAF > 25%) genetic variants. **b** An example of common haplotypes inside the segment 23 of Chromosome 1. Haplotypes were constructed from 50 adjacent high-frequency (MAF > 25%) genetic variants and are represented by the strings of fifty 0 s and 1 s, where “0” means the presence of a reference allele, while “1” means an alternative allele in the haplotype. The haplotypes that occur ≥100 times in the 1092 individuals are defined as ‘common haplotypes’ and are listed in descending order of their occurrence. The exemplified segment 23 has three common haplotypes. Putative BGC events were searched only in individuals who have one common haplotype and another nearly identical rare haplotype, which has only one allele difference with the common haplotype at the “acceptor” site (marked with a blue square). In this example such conditions were found in individual NA20787 from the TSI population. In the two parental haplotypes of TSI_NA20787, the first (Parent 1) is a common haplotype, which occurs 216 times in the 1092 genomes. The other haplotype (Parent 2), despite being identical to the common haplotype at 49 polymorphic sites, is a rare haplotype which occurs only once in the 1092 individuals. This rare haplotype contains the Acceptor site (marked with a blue square), which represents a case of putative base pair conversion event at this location in one of the ancestors of this individual. The location of this acceptor site in the haplotype string, its reference and alternative alleles, and base change due to BGC event (purple arrow) is shown in the bottom of the figure. Detailed information about every segment and all putative BGC events are available from our web site

On the other hand, Glemin and co-authors used analysis of derived allele frequency from the 1000 Genomes data to quantify gBGC in human [32]. We chose another approach for detection of relatively recent SNP flip-overs, due to gBGC that happened hundreds or thousands years ago. We computationally analyzed only common haplotypes built from frequent SNPs that remained unchanged in different populations for thousands of years. Then, we looked for a very rare haplotype that is practically identical to one of the common haplotypes except with one allele replacement (flip-over) at one of the polymorphic sites in the middle of the haplotype. We called this specific type of rare haplotype as “Acceptor” haplotype (See Fig. 1). We searched 1092 sequenced genomes for people that have the acceptor haplotype from one parent and its nearly identical common haplotype counterpart inherited from another parent (as illustrated in Fig. 1b). This requirement for parental haplotype organization is important for avoiding possible errors due to low sequencing coverage in the 1000 Genomes dataset. Indeed, under such conditions all polymorphic sites within a haplotype under analysis are homozygous except the one “Acceptor” site representing putative gBGC conversion event. This homozygosity requirement eliminates possible phasing errors.

In this paper, we performed bioinformatics investigation of SNPs in the “1000 Genomes” database and observed 223,955 putative cases of gBGC events. We report here a quantitative assessment of the aftermath of gBGC in.

## Methods

Genotype datasets for all the human chromosomes of the 1092 human genomes were downloaded from the 1000 Genomes ftp site (ftp://ftp-trace.ncbi.nih.gov/1000genomes/ftp/release/20110521/) [33] as Variant Call Format (VCF) files version 4.1 [34]. This database contains a total of 38.2 M SNPs, 3.9 M short indels and 14 K deletions for all the human chromosomes that have been used in this study. Information about parental haplotypes has been taken directly from Phase 1 of 1000 Genomes Project, since its genomic sequences were entirely “phased”. We considered only bi-allelic variants for simplicity and because we had sufficient statistics in our datasets. All multi-allelic variants were skipped. Allele frequency for every SNP was obtained from the “AF=” field inside column 8 of the 1000 Genome VCF files. We did not take into account differences in population frequencies in our paper because this frequency variation is not relevant at all for finding SNP flip-over events, which is the major focus in this study.

All haplotypes of 1092 individuals, putative cases of gene conversion and local GC content of putative gene conversion sites were obtained with our pipeline of five Perl programs (*HaploFind.pl, GeneConversionFind.pl, BGC_Calculator.pl, LocalGC_calculator.pl, AT_vs_GC.pl, RandomGC_Calculator.pl*). Detailed description and scripts of all our Perl programs, their instruction manuals, the command lines for execution of programs, and examples of output files can be found in the Additional file 1.

Haplotypes for every chromosomal segment were computationally constructed from 50 adjacent frequent genetic variants (MFA > 25%). Since more than 90% of these frequent genetic variants are presented by SNPs, we call these variants as SNPs for simplicity throughout the text. However, for putative BGC events having “flip-over” alleles inside common haplotypes we considered only SNPs (all indels were rejected from consideration).

Our computer characterization of common haplotypes has been elaborated in the predecessor project [35]. In that research we tried different parameters for the threshold for frequent SNPs (Minor allele frequency (MAF) 10%, 20, 25, or 30%) and the number of frequent SNPs in the haplotypes (30, 50, or 70). In the present paper we chose the default parameters of the previous research (MAF = 25%, number of SNPs in the haplotype = 50), which allow identifying an optimal amount of common haplotypes. The number of common haplotypes and the linkage disequilibrium between SNP within a common haplotype varies from one chromosomal segment to another as described in Dutta et al., [35].

The exact of base pair changes from Common haplotype to Acceptor haplotype have been characterized by the Perl script *GeneConversionFind.pl.*

Statistical analyses were performed using *R* package [36]. One sample T-test was used to calculate the standard deviations for the occurrence of common haplotypes. The standard errors for the ‘AT ➔ GC’ and ‘GC ➔ AT’ events were calculated with the formula using the Rule of Sample Proportions

$$ \mathbf{SE}=\sqrt{\frac{\mathbf{p}\left(\mathbf{1}\hbox{-} \mathbf{p}\right)}{\boldsymbol{N}}} $$

Where N is total number of gene conversion events (AT ➔ GC and GC ➔ AT), p is the proportion of AT ➔ GC events and (1 – p) is the proportion of GC ➔ AT events.

All our programs are freely available from our website (http://bpg.utoledo.edu/~afedorov/lab/BGC.html) [37]. The entire dataset of all haplotypes for each 56,328 chromosomal segments generated by our programs is also available from this web site (this dataset is too big to place it in Additional file 1).

## Results

### Chromosomal segments and common Haplotypes (CHs)

All human autosomes were divided into 56,328 consecutive segments with a default size of 500 kb as illustrated in Fig. 1a. A complete list of segments for all chromosomes is presented in Table 1. For each chromosomal segment, we determined SNP haplotypes built from 50 adjacent genetic variants occurring with high frequency (Minor Allele Frequency > 25%) in 1092 individuals. Each of 1092 individuals from phase 1 of the 1000 Genomes Project is represented by two haplotypes that correspond to the two parents of the sequenced individual. All 2184 haplotypes were ranked by their occurrences as explained in Fig. 1b. When a haplotype was found 100 or more times among 1092 studied individuals, it was considered a *Common Haplotype* (CH). The program *HaploFind.pl* automatically lists all common and rare haplotypes among all autosomes in 56,328 segments. The average size of our haplotypes (47.8 kb) is congruent with the findings of Gabriel and co-authors [38]. They found that most of the human genome can be divided into blocks/segments of substantial size and, within each of them, very few common haplotypes capture a vast majority (~ 90%) of the chromosomes in each population.

**Table 1:** Distribution of computational segments and putative gene conversion events in all autosomes

Chromo-some	# of Segments	Rare haplotype count = 1				Rare haplotype count <= 5			
		AT → GC cases	GC → AT cases	No Base Change cases	Total cases	AT → GC cases	GC → AT cases	No Base Change cases	Total cases
1	4359	1964	1677	637	4278	8973	8200	3181	20,354
2	4660	2318	1921	760	4999	10,514	8928	3605	23,047
3	4048	1977	1640	632	4249	8785	7715	3196	19,696
4	4191	1945	1891	722	4558	9034	8442	3373	20,849
5	3687	1786	1513	629	3928	7842	7190	3052	18,084
6	3869	1791	1614	629	4034	8516	7835	2975	19,326
7	2838	1361	1227	492	3080	6133	5431	2311	13,875
8	3179	1631	1287	560	3478	7124	6170	2691	15,985
9	2389	1163	963	420	2546	4898	4253	1814	10,965
10	2830	1311	1112	423	2846	5897	5283	1997	13,177
11	2838	1352	1196	479	3027	5943	5444	2302	13,689
12	2676	1150	1035	391	2576	5475	4978	1953	12,406
13	2135	948	772	282	2002	4334	4000	1518	9852
14	1852	862	757	277	1896	3781	3368	1422	8571
15	1647	789	627	251	1667	3362	2822	1176	7360
16	1723	794	629	367	1790	3440	2787	1501	7728
17	1540	713	606	201	1520	3144	2709	1027	6880
18	1629	720	603	226	1549	3057	2728	1110	6895
19	1334	662	511	196	1369	2678	2314	868	5860
20	1272	559	449	146	1154	2350	2083	785	5218
21	840	384	326	121	831	1620	1411	574	3605
22	792	403	290	125	818	1643	1321	523	3487

The first two columns of Table 1 lists the number of computationally generated segments in different human autosomes. The next four columns describe number of AT to GC, number of GC to AT, number of ‘No Base Change’ and total mismatch repair cases respectively in all autosomes when only single rare haplotype occurrence in the 1092 genomes was considered. The last four columns present number of AT to GC cases, number of GC to AT cases, number of ‘No Base Change’ cases and total mismatch repair cases respectively in all autosomes when rare haplotype occurrence <= 5 in the 1092 genomes was considered

### Putative gene conversion events

To test the gBGC hypothesis, we identified the putative cases of nucleotide changes (SNP flip-overs) due to gene conversion events as illustrated in Fig. 1b using Perl script *GeneConversionFind.pl*. The script *GeneConversionFind.pl* identifies individuals who have a common haplotype inherited from one parent and another almost identical (49 matching alleles out of 50) but rare haplotype (occurrence ≤5 times among 1092 individuals) inherited from another parent. In the rare haplotype, the only site, which is not identical to the common haplotype, has a SNP flip-over (replacement of an allele inherited from one parent by its complementary allele inherited from another parent). We named this polymorphic site an ‘Acceptor site’, and named the rare haplotype an ‘Acceptor haplotype’. Such cases of SNP flip-overs represent putative gene conversion events, which may have occurred in the genomes of parents of the analyzed individuals or in their genetic predecessors. We have considered only those cases where the ‘Acceptor’ haplotype occurs once, twice, and ≤ 5 times in the 1092 Genomes population. For each case, the program *HaploFind.pl* identifies the Acceptor site, notes its location in the Acceptor haplotype, reference allele and alternative allele at the Acceptor site, and also computes the type of putative gene conversion event at that particular site. A portion of a representative output file generated by the program is shown at the bottom (right side) in Fig. 1b.

**Fig. 2:**
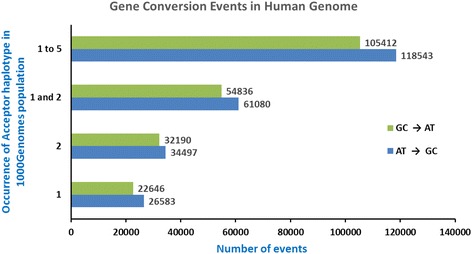
Number of AT - > GC vs GC - > AT changes due to putative base pair conversion events. The number of identified base pair conversion events is presented along the horizontal axis, while the vertical axis shows the different computational conditions for registration of these events. We considered cases where the rare haplotype (with the Acceptor site) occurs only once among the 1092 individuals (labeled as 1, at the bottom), twice among the 1092 individuals (labeled as 2), single and double occurrences taken together (labeled as 1 and 2) and less than or equal to 5 occurrences among the 1092 individuals (labeled as 1 to 5)

The next Perl script *AT_vs_GC.pl* computes occurrence of different types of SNP flip-overs (AT- > GC or GC- > AT). The program *BGC_Calculator.pl* was used to combine and summarize results for different chromosomes together and to generate final results, which are shown in Fig. 2.

When we considered cases where Acceptor Haplotype occurs only once in a person from the 1092 Genomes populations, we found 26,583 AT- > GC SNP flip-overs and noticeably less (22,646) GC- > AT SNP flip-overs. A two-tailed Chi-square test was performed with these numbers and gave us a *p*-value < 10^− 16^, which is statistically significant. When we registered Acceptor haplotype only once, it may be interpreted as sequencing error. However, occurrence of the same Acceptor haplotype in more than one individual reduces the possibilities of such false positives. Therefore, in addition, we identified all SNP flip-over cases where the same acceptor haplotype was found in 2 to 5 individuals (see Fig. 2). We observed similar trends with varying Acceptor haplotype occurrences. This fact testifies that sequencing errors cannot be the reason for our observations.

When the Acceptor Haplotypes which occurred two times among the 1092 individuals were considered, 34,497 and 32,190 cases of AT- > GC and GC- > AT conversion events were found respectively. When Acceptor Haplotypes which occurred in less or equal to five persons were considered, 118,543 AT- > GC conversions were identified compared to 105,412 cases of GC- > AT conversions. Chi-square tests performed in each case above resulted in a p-value < 10^− 16^, which shows the statistical significance of the results. All in all, we report a 11.7% bias in AT - > GC over the GC- > AT SNP flip-overs.

### Local GC content

**Fig. 3:**
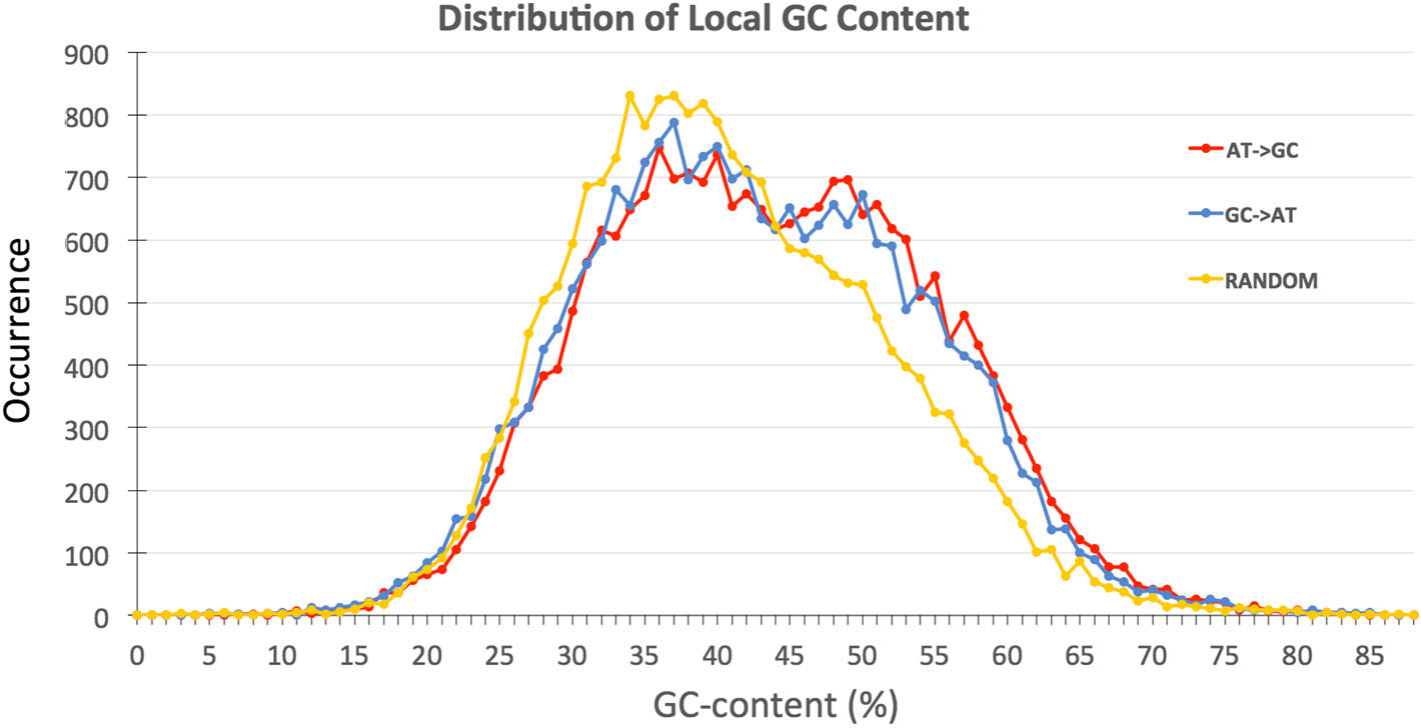
Distribution of local GC content in regions surrounding AT - > GC vs GC - > AT conversion events. Local GC content was calculated within 100 bp window by considering 50 nucleotides before and after each putative AT - > GC or GC - > AT conversion site The red line shows the distribution of local GC content around 22,646 AT - > GC conversion events while the blue line presents the local GC content around the same number of GC - > AT cases. The yellow line (control) represents local GC content around same number of sites selected randomly, independent of gene conversion events.

Distribution of GC-content at the sites of the conversion events is an important consideration in the BGC hypothesis. Therefore, we calculated local GC content within 100 nucleotide-long window considering 50 nucleotides before and after each putative AT - > GC or GC - > AT conversion site. Our laboratory had previously developed an approach to evaluate various local biases in nucleotide composition and intensively studied non-randomness of such local nucleotide compositions in the human genome [39, 40]. In these examinations 100 nucleotide-long scanning window had been chosen as a default parameter in these genomic sequence analyses. Thus, we keep the same window size in this project. We used the program *LocalGC_calculator.pl* to calculate local GC content around 22,646 AT- > GC conversion events and same number of GC- > AT sites. Another program *RandomGC_Calculator.pl* was used to calculate local GC-content around 22,646 randomly selected sites, which served as a control for the overall distribution of GC-rich regions. These results are presented in Fig. 3. It is well-established that recombination is more frequent in GC-rich regions [15], and consequently BGC events should occur more frequently in GC-rich regions of the genome. The distribution of local GC-content around putative BGC sites obtained from our computations confirms this conjecture. Indeed the rate of both AT- > GC and GC- > AT SNP flip-overs in GC-rich regions is about 22% more frequent than the random expectation. We performed Chi-squared test of Goodness-of-Fit with the null hypothesis that there is no difference between the expected distribution of GC content (distribution around random sites) and the observed distributions (distributions of Local GC content around the AT- > GC and GC- > AT sites). The *p*-value for the test comparing AT- > GC and random distributions was < 2.2 × 10^− 16^. The test comparing GC- > AT and random distributions also resulted in a p-value < 2.2 × 10^− 16^. These extremely low *p*-values show that the null hypothesis is not true and both the observed distributions of GC content are significantly different than the distribution of GC content around random sites. We did not see a significant difference between the distribution of local GC-content for AT- > GC events versus GC- > AT events. Our results confirm that, in AT-rich regions (GC-composition < 40%), there are ~ 10% fewer events of gBGC base pair flip-over compared to random expectation (blue and red lines are ~ 10% lower than yellow line on the left side of Fig. 3). In contrast, in GC-rich regions (GC composition > 44%), there are ~ 25% more putative gBGC base changes over the random expectation (blue and red lines are higher than yellow line on the right side of Fig. 3). These results are in line with gBGC theory. However, the detected disparity is only moderate (10–25% difference from random distribution on Fig. 3).

## Discussion

What is the average number of SNP flip-overs in a human gamete? The estimation of this number is essential because SNP flip-overs change SNP-haplotypes and linkage disequilibrium between SNPs. This effect should be taken into consideration in various programs used for deciphering phenotypes from SNP patterns because noticeable SNP flip-over process constantly modifies these patterns and reduces linkage disequilibrium between neighboring SNPs. SNP flip-over occurs during NCO meiotic recombination events when one allele is replaced by its counterpart allele, while the neighboring SNPs in the haplotype remain the same. NCOs are up to 15 times more frequent than COs [41–45]. A recent study estimated 228 NCO events on average per generation in humans [27]. At the same time, average length of NCO heteroduplex tracts are much shorter than CO tracts with average NCO tract length of 75 bp according to several recent studies [27, 44, 46]. Therefore, we estimate 228 × 75 bp = 17.1 kb of total NCO heteroduplex length per gamete. On the other hand, the number of heterozygous sites for Europeans and Asian individuals in the 1000 genomes dataset are about 2.3 million, and about 3.3 million for African individuals [34]. Taking these groups together, on average there is about one heterozygous site per 1.2 kb in the human genome. Considering all the above, in human meiosis, about 14.2 mismatches (17.1 kb/1.2 kb) should be formed within all NCO heteroduplexes of a gamete. During repair, only half of these 14.2 mismatches should resolve into SNP flip-overs, while in the other half of cases, MMR should restore the original alleles within original haplotypes. This leaves 7.1 SNP flip-overs per gamete. They represent up to a quarter of all new mutations in a gamete (there are from 20 to 50 novel mutations in a gamete according to different estimations [47–49]). Hence, SNP flip-over has a substantial impact on nucleotide changes and should be considered in any SNP dynamics analyses.

The second important question we address is the number of AT ➔ GC versus GC ➔ AT base-pair conversions per human gamete due to gBGC. To estimate this number, we should consider both CO and NCO cases since both results in base pair conversions. The estimated sex-averaged number of COs per generation is ~ 30 [50]. We will use the average CO heteroduplex tract length of 600 bp for humans, which is consistent with several current studies [46, 51]. So, we estimate in total 30 × 600 bp = 18 kb of CO heteroduplex tracts length per gamete. Thus, number of mismatches formed in all CO heteroduplexes during human meiosis is about 15 (18 kb / 1.2 kb). In the previous paragraph we already calculated that the average number of mismatches in NCO heteroduplexes is 14.2 per gamete. Since, half of mismatches should be resolved in base-pair changes, the total number of base-pair changes due to both NCO and CO will be, on average, 14.6 events per gamete. According to our calculations (Table 1), 15.4% of SNP flip-overs do not cause AT/GC base pair changes, 45.7% create AT- > GC base pair replacements, while 38.9% are responsible for the reverse GC- > AT replacements. Thus, on average, 14.6 base-pair change events should generate 2.2 cases with no GC/AT changes (i.e., GC < −>CG and AT<− > TA), 6.7 cases with AT- > GC base pair conversions, and 5.7 cases of GC- > AT conversions. In sum, every human gamete should generate an excess of 1 AT- > GC base pair changes due to gBGC episodes. This number is twice the estimates for yeast genomes [14]. At the same time, our assessment is lower than that of Williams and coauthors who evaluated that 68% heterozygous AT/GC SNPs transmit GC alleles [27]. The overabundance of 1 AT- > GC base pair changes per gamete, if occurring over millions of years in mammals, may yield significant biases in GC-compositions along chromosomes. However, this effect should be evaluated only in combination with an influx of de novo mutations, which is roughly 20–50 mutations per gamete [47–49]. Thus, overabundance of 1 AT- > GC base changes per gamete due to gBGC represents only 2–5% of all new mutations in the gamete genome and might be over-shadowed by other mutational processes. Distribution of novel mutations is uneven along the genome and depends on the local nucleotide composition at the site of mutations. Our laboratory reported a strong fixation bias favoring AT - > GC mutations in GC-rich regions in humans and the opposite fixation bias favoring GC - > AT mutations in AT-rich regions and other fixation biases (e.g. Pu - > Py in pyrimidine rich regions) [40]. Therefore, estimation of the total effect of mutations and conversions on the genomic GC composition is very intricate and still awaits thoughtful modeling.

## Conclusions

During the process of meiotic non-crossover recombination, a human gamete acquires about 7 SNP flip-over events, in which one allele is replaced by its complementary allele while the neighboring SNPs in the haplotype remain the same. On an average, GC-Biased Gene Conversion increments the GC-content by substitution of one AT pair by one GC pair in every haploid human genome. Happening over millions of years of evolution, this smallest bias may be a noticeable force in changing the nucleotide composition landscape along chromosomes.

## Additional file

Additional file 1: Instruction manual for Perl programs for testing BGC hypothesis. This file contains detailed instructions and protocols of Perl programs for construction and analysis of haplotypes of frequent genetic variants. (DOCX 334 kb)

## Abbreviations

CH: Common haplotype
CO: Crossover
gBGC: GC-biased gene conversion
MAF: Minor allele frequency
MMR: DNA Mismatch Repair
NCO: Non- crossover
SNP: Single nucleotide polymorphism
