# Supplementary material for: 1000 human genomes carry widespread signatures of GC biased gene conversion
Source: BMC Genomics. 2018 Apr 16;19:256. doi: 10.1186/s12864-018-4593-1 (PMC5902838; doi:10.1186/s12864-018-4593-1)
Supplement: Supplementary file 1 — Instruction manual for Perl programs for testing BGC hypothesis. This file contains detailed instructions and protocols of Perl programs for construction and analysis of haplotypes of frequent genetic variants. (DOCX 334 kb) [file 12864_2018_4593_MOESM1_ESM.docx]

**Instruction manual for Perl programs for testing BGC hypothesis**

**Human Chromosomes and 1092 Individuals:**

The 1000 Genome Phase I database contains SNP data for all 22 human autosomes and the ‘X’ chromosome for each of the 1092 individuals from 14 different populations belonging to four different continents. Version 4.1 of this dataset contains a variety of genetic variants (GVs) - a total of 38.2M SNPs, 3.9M short indels and 14K deletions for all the human chromosomes. Since the majority of the GVs are SNPs, here we will use the word SNP for all GVs.

**The 1000Genomes data files in INTRON2:**

The 1000Genomes data files are stored in a specific directory of the supercomputer intron2 workstation (refer figure 1 below).


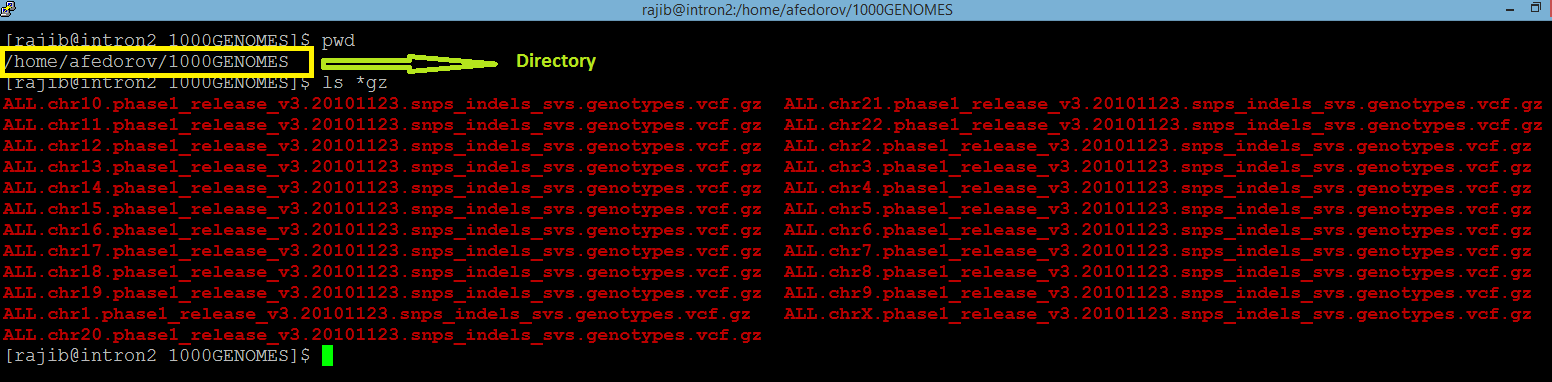


**Figure 1: screenshot of directory with 1000Genomes data files in the supercomputer intron2 workstation**

**STEP I: Extraction of SNPs and building the haplotypes:**

The perl script ***Haplo_find.pl*** opens and processes the 1000 Genomes .vcf.gz file for the desired chromosome. This program filters the SNPs with desired frequency and processes them into haplotypes. The user chooses the parameters (such as the chromosome of choice, desired frequency of SNPs, number of SNPs in the haplotype and a threshold count for defining the Common haplotypes) according to preferences in the arguments in the command line. For example, let us consider the following screenshot of a command line for executing ***Haplo_find.pl****:*


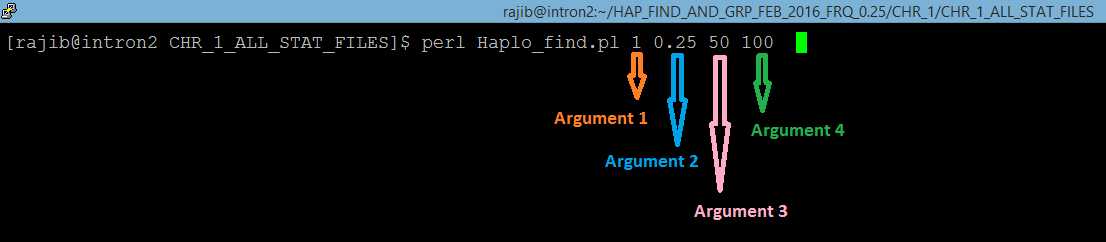


**Figure 2: screenshot of command line for execution of Haplo_Find.pl**

The command line in the figure 2 above computes the haplotypes for Chromosome **1 (argument 1)**, with SNPs having frequency >**0.25** **(argument 2)** for minor alleles (MAF). The program constructs haplotypes with **50 (argument 3)** such adjacent frequent SNPs. Common haplotypes will be those which occur >=**100 (argument 4)** times in the populations (1092 individuals).

While processing the .vcf.gz file for the intended chromosome, the script ***Haplo_find.pl*** considers the first SNP coordinate in the chromosome as the beginning of the first **SEGMENT**. In the 1000Genomes dataset, the SNP coordinate is in the second column under the header ‘POS’ and the SNP ID is in the 3^rd^ column with ‘ID’ header (see screenshots below). The program starts scanning the file for SNPs with user specified frequency (>0.25 MAF in the given example). The frequency is denoted as AF and provided in the INFO column (column #8, see figure 3 below).


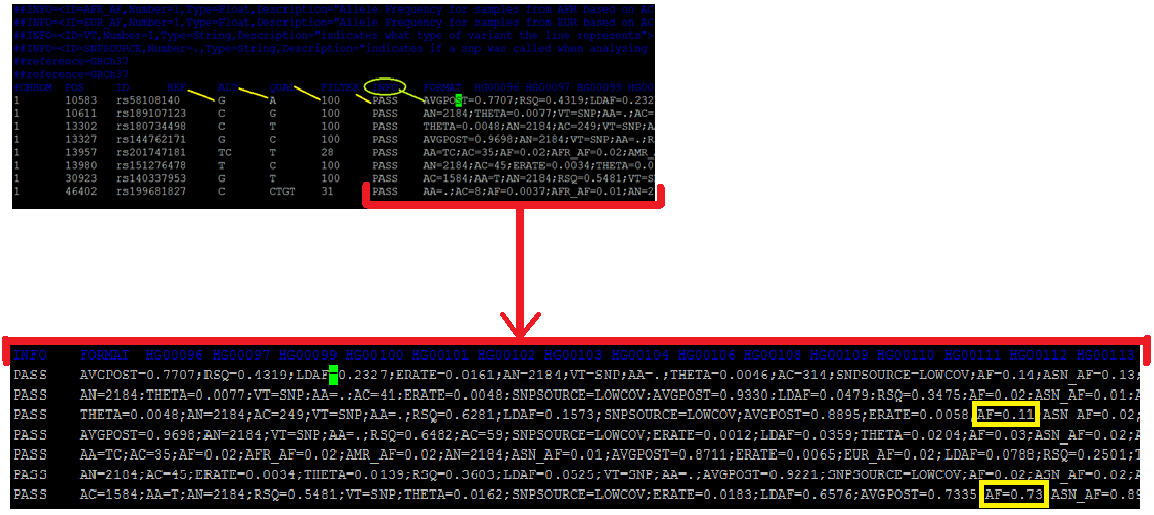


**Figure 3: screenshots of 1000Genomes vcf.gz file format**

When the program finds the first SNP with the desired frequency, it notes the coordinate and keeps looking for the desired SNPs until it finds the 50^th^ such SNP. When the 50^th^ SNP is found, the coordinate of the 50^th^ SNP is marked. The distance between the 1^st^ and the 50^th^ SNP is defined as the **HAPLOTYPE LENGTH**. The next segment starts from the very next SNP.

The 50 lines corresponding to the 50 filtered SNPs, containing the general information and genotype information of the 1092 individuals are saved in output files named **ALL_STATS_Cx-y** (where x denotes the Chromosome number and y denotes the segment number). The genotype information of the 1092 individuals are then extracted by the program.

Genotype annotations for an individual homozygous with mutant genetic variants is annotated 1|1 in the VCF file. Homozygous presence of reference alleles in an individual is annotated 0|0. Genotype of heterozygous individual is annotated 0|1 or 1|0. These genotype information is available from the column respective to each individual identified by a unique identifier (HGxxxxxx or NAxxxxxx) in the header line. The figure 4 below shows these genotype information and Individual Identifiers from one of the .vcf.gz files. Since the 1000Genomes data is phased, we have denoted the first genotype as M (maternal) and the second as P (paternal) for ease of understanding, although practically it is not possible to identify the maternal and paternal chromosome with certainty from this data.


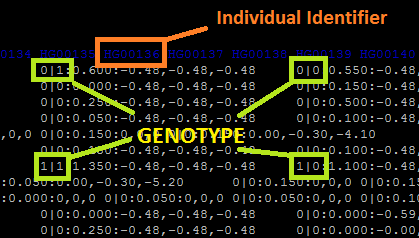


**Figure 4: screenshot of genotype annotation in one of the 1000Genomes data files**

The program Haplo_Find.pl extracts and stores these genotype information for each of the 1092 individuals for the 50 SNPs of interest (MAF >0.25) and constructs the two haplotypes (maternal and paternal) for each of the 1092 individuals. Practically, each haplotype is a string consisting of 50 single digits- 0s and 1s.


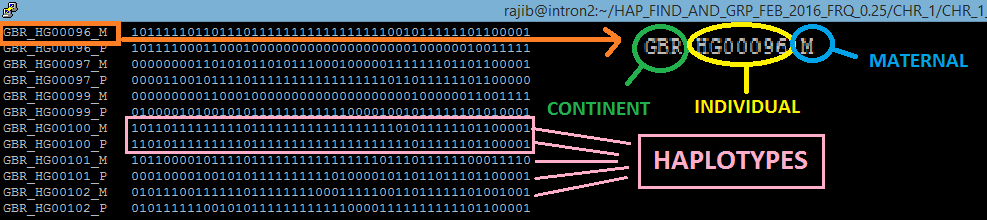


**Figure 5: screenshot showing haplotypes in one of the output files named ALL_STATS_Cx-y**

From the .vcf file, the program also extracts the individual identifier. The population each individual comes from is available from the 1000Genomes website. These two information are then combined together by the program to create a unique identifier along with M (for the maternal chromosome) and P (for the paternal chromosome). Thus, for example, in the figure 5 above, two chromosomal strands for the individual highlighted are denoted as GBR_HG00096_M and GBR_HG00096_P where GBR is the population and HG00096 is the individual identifier. M and P stands for maternal and paternal respectively.

The haplotypes are stored along with the respective identifiers in the output files named **ALL_STATS_Cx-y** (where x denotes the Chromosome number and y denotes the segment number).

Finally, the algorithm compares the 2184 haplotypes (strings of 0s and 1s as shown in the figure above, 2 * 1092 = 2184) with each other and counts the occurrences of the haplotypes. The haplotypes that occur more than the user specified threshold (in the example here >=100) are defined as Common haplotypes. These common haplotypes are listed at the bottom of the output file **ALL_STATS_Cx-y** (see figure 6 below).


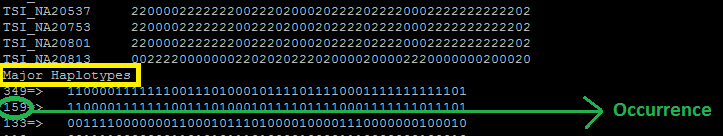


**Figure 6: screenshot showing Major haplotypes in one of the output files named ALL_STATS_Cx-y**

These processes continue for the entire length of the chromosome and the resulting segments, their haplotypes and other information are saved in automatically created output files for each respective segment.

This STEP requires about 15 hr to finish computation for Chromosome 2 which is one of the largest chromosomes and have the highest number of SNPs with frequency >0.25 (233,023). Therefore, for each of the other chromosomes, complete execution of this program requires less than 15 hr.

This program was created for using in another project in our lab. The manuscript of that project is currently under review. Therefore this program is capable of generating several different output files according to user preference. For the current project, we have chosen to generate only one output file for each chromosomal segment. Therefore, upon execution, this program automatically generates the following output files:

1. **ALL_STATS_Cx-y** for all segments. For example:

**% vi ALL_STATS_C1-143**

This output file was created on Sun Mar 13 00:08:06 2016

CHR_1 Segment_143 Hap Length= 49259

Total no. of SNPs in this segment= 706

SNPs with freq > 0.25 & < 0.75 are:

1 71320836 rs34747258 GA G 442 PASS AA=GA;AC=605;AF=0.28;AFR_AF=0.22;AMR_AF=0.30;AN=2184;ASN_AF=0.20;AVGPOST=0.9943;ERATE=0.0006;EUR_AF=0.36;LDAF=0.2768;RSQ=0.98

1 71325044 rs1327460 T C 100 PASS ERATE=0.0004;AVGPOST=0.9985;AA=C;AC=1376;AN=2184;VT=SNP;LDAF=0.6295;THETA=0.0006;SNPSOURCE=LOWCOV;RSQ=0.9975;AF=0.63;ASN_AF=0

**STEP II: IDENTIFYING THE PUTATIVE GENE CONVERSION EVENTS**

This step uses a system call (**system_for_BGC.pl)** to run two Perl scripts ***GeneConversionFind.pl*** and ***AT_vs_GC.pl*** for all the segments created for each chromosome in STEP I.

The command line for execution of this step for chromosome 1 is as follows:

**% perl system_for_BGC.pl 1 4660**

The *Perl* script ***GeneConversionFind.pl*** uses the files named **ALL_STATS_Cx-y** generated in step 1 as inputs for each segment. The program scans the input file for each segment and identifies the common haplotypes for that segment. Next, the program finds individuals who have 1 common haplotype and another nearly identical (49 similarities out of 50 SNPs) rare haplotype (occurs <=5 times in 1092 individuals and occurrence of rare haplotype can be altered according to user preference). The program identifies the position where the individual is heterozygous, i.e. the position where the two haplotypes differ. Then the program takes note of the reference allele and alternative allele of that position and computes the type of putative gene conversion event that might have occurred at this position in one of the previous generations of this individual. The detailed results are saved in an output file named **Gene_Conversion_events_C_x**, where x is the chromosome number. A picture of a portion of the file **Gene_Conversion_events_C_1** is provided below as an example.


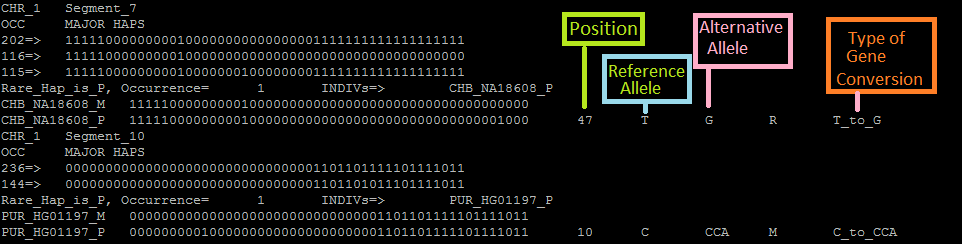


**Figure 7: screenshot showing a portion of the file Gene_Conversion_events_C_1**

Another output for each chromosome file is automatically created where only the summary of the results are saved. This file is named **Gene_Con_Summary_C_x**, where x is the chromosome number. A screenshot of the **Gene_Con_Summary_C_1** file is given below which contains the summary for the same portion shown in the example above.


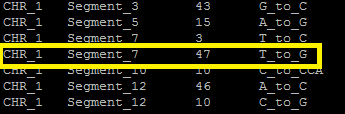


**Figure 8: screenshot showing a portion of the file Gene_Con_Summary_C_1**

The Perl script AT_vs_GC.pl uses this file for each chromosome as input files and calculates the occurrence of various types of putative gene conversion events in each chromosome separately. This script generates the output file named **Gene_Con_Occurrence_List_C_x,** where x is the chromosome number. This output file lists all the different gene conversion events along with their occurrence. An example from **Gene_Con_Occurrence_List_C_1** is given below:


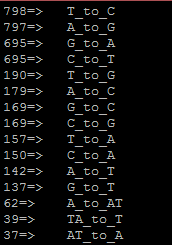


**Figure 9: screenshot showing a portion of the file Gene_Con_Occurrence_List_C_1**

We can see that in chromosome 1, 798 T to C gene conversion events were found while 797 A to G events and 695 G to A events were identified and so on for other different types of gene conversion events.

In addition to the above files, two other output files are generated for each chromosome in this step. These are **Stat_For_GC_Content_AT_to_GC_C_x** and **Stat_For_GC_Content_GC_to_AT_C_x,** where x is the chromosome number. A screen shot of the file named **Stat_For_GC_Content_AT_to_GC_C_1** is given below.


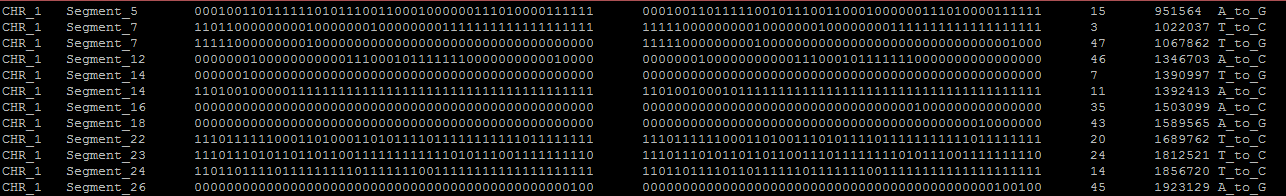


**Figure 10: screenshot showing a portion of the file Stat_For_GC_Content_AT_to_GC_C_1**

**STEP III: Calculating the GC bias**

The short Perl program ***GC_Bias_calculator.pl*** is used to calculate the total occurrences of different AT to GC and GC to AT conversion events. This program uses the **Gene_Con_Occurrence_List_C_x** files (generated in STEP II) as input files and performs all calculations relevant to find any existing bias in the AT to GC and GC to AT gene conversion events. All other events which include AT to AT or GC to GC (No base pair change) events, insertions, deletions are ignored for the final summary calculations. These results are saved in an output file named GeneConv_Bias_Results_Summary. Another output file is also created at the same time where the summary statistics for each chromosome is saved separately. This file is named **GeneConv_Bias_Results_Rare_Hap_Occ_1_cont_Separate_CHR.** This file is transferred to the PC/Mac environment for further analysis and visual presentation of results.

**STEP IV: Local GC content calculation**

To calculate the local GC content, two Perl scripts were used. The first Perl script **BGC_LOCAL_GC_content_calculator_Final.pl** Two output files created in STEP II (named **Stat_For_GC_Content_AT_to_GC_C_x** and **Stat_For_GC_Content_GC_to_AT_C_x**, where x is the chromosome number) are used as input files by this program to identify the coordinates of the putative AT to GC and GC to AT conversion sites. The 6^th^ column of both these files contains the coordinates of all the putative gene conversion sites (see Fig 10 for example). This program then scans the respective Denisovan chromosome which contains the nucleotide composition of the reference Human genome. The program extracts 50 nucleotides before and 50 nucleotides after each putative gene conversion site and calculates local GC content. The output files generated are **GC_CONTENT_GC_to_AT_stat_C_x** and **GC_CONTENT_AT_to_GC_BIN_C_x,** where x is the chromosome number. Files named **GC_CONTENT_AT_to_GC_BIN_C_x** were further processed by the Perl script **GC_single_Bins_explorer_final.pl** to obtain final results which are stored in the output file named **BINS_single_results_Rare_Hap_1_final.** This file was transferred to the PC/Mac environment for further analysis and presentation of results.

Another program **Local_GC_random.pl** was used to calculate local GC content of random sites in the human reference genome.
